# Evaluation of the Relationship between the NRT-Ratio, Cochlear Anatomy, and Insertions Depth of Perimodiolar Cochlear Implant Electrodes

**DOI:** 10.1155/2015/706253

**Published:** 2015-12-29

**Authors:** Philipp Mittmann, Grit Rademacher, Sven Mutze, Frederike Hassepass, Arneborg Ernst, Ingo Todt

**Affiliations:** ^1^Department of Otolaryngology, ukb, 12683 Berlin, Germany; ^2^Department of Radiology and Neuroradiology, ukb, 12683 Berlin, Germany; ^3^Department of Otolaryngology, University of Freiburg, 79106 Freiburg im Breisgau, Germany

## Abstract

The position of the cochlear implant electrode array within the scala tympani is essential for an optimal postoperative hearing benefit. If the electrode array changes in between the scalae intracochlearly (i.e., from scala tympani to scala vestibuli), a reduced auditory performance can be assumed. We established a neural response telemetry-ratio (NRT-ratio) which corresponds with the scalar position of the electrodes but shows within its limits a variability. The aim of this study was to determine if insertion depth angle or cochlea size influences the NRT-ratio. The intraoperative electrophysiological NRT data of 26 patients were evaluated. Using a flat panel tomography system, the position of the electrode array was evaluated radiologically. The insertion depth angle of the electrode, the cochlea size, and the NRT-ratio were calculated postoperatively. The radiological results were compared with the intraoperatively obtained electrophysiological data (NRT-ratio) and statistically evaluated. In all patients the NRT-ratio, the insertion depth angle, and the cochlea size could be determined. A significant correlation between insertional depth, cochlear size, and the NRT-ratio was not found. The NRT-ratio is a reliable electrophysiological tool to determine the scalar position of a perimodiolar electrode array. The NRT-ratio can be applied independent from insertion depth and cochlear size.

## 1. Introduction

Cochlear implantation (CI) is a safe and effective procedure for patients with residual hearing and profound sensorineural hearing loss (SNHL). The insertion depth of cochlear implant electrode arrays has been described to correlate with preservation of residual hearing and word identification scores [1–5]. If the electrode array changes from the scala tympani to the scala vestibuli, a poorer audiological outcome is most likely [6, 7]. It is reported that an insertion of a CI electrode array into the scala tympani results in a better postoperative speech perception compared to a position of the electrode in the scala vestibuli [8, 9]. Translocation of electrode arrays from the scala tympani into the scala vestibuli is known to occur in most cases at an electrode insertion depth of approximately 180° [10].

Postoperative standard X-ray can be used to determine the insertion depth [11, 12] but different imaging techniques such as computed tomography (CT), flat panel tomography (FPT), or digital volume tomography (DVT) are needed to verify the electrode's scalar position [8]. Intraoperative 3D rotational X-ray can be used to produce high quality, real-time images of the cochlea and the electrode array in the operating room [8, 13]. Nevertheless, this technique is time and cost consuming but provides reliable evidence about the intracochlear position of the electrode array.

Another way to verify the electrode array's scalar position is the evaluation of intraoperative electrophysiological measurements. The spread of excitation measurements can identify electrode array fold-overs [14]. Cochlear implant devices from Cochlear Ltd. are equipped with the neural response telemetry (NRT) system, which can measure the electrically evoked whole nerve potentials (EAP) without accessing the cochlear directly [15]. The EAP depends on the distance between the electrode array and the spiral ganglion [16]. For the perimodiolar Nucleus Contour electrode arrays an apical to basal neural response telemetry-ratio (NRT-ratio) can be used to determine the intracochlear position of the electrode array [17, 18]. A NRT-ratio above 1.05 indicates an electrode translocation from scala tympani to scala vestibuli and a NRT-ratio below 1.05 shows correlation to a pure scala tympani placement [17]. But within the group of patients with an electrode placement within scala tympani a variation of the NRT-ratio was seen [17, 18].

It was therefore the aim of the present study to investigate the dependence of the NRT-ratio of the electrodes on insertion depth angles and cochlea sizes in a group of patients with radiologically confirmed scalar tympani position.

## 2. Materials and Methods

A total of 26 patients were included in this retrospective study. The inclusion criterion was the implantation with a Nucleus Contour Advance electrode. All included patients were implanted by the senior author between 2010 and 2015 with a standard surgical procedure including a postauricular transmastoid approach, a posterior tympanotomy, and a round window or extended round window access, as well as the AOS technique for electrode insertion. Furthermore, all of the included patients showed stable intraoperatively measured t-NRT sweeps and a postoperative radiologic evaluation of the electrode's position based on a rotational tomography (RT) with a digital flat panel detector. The study was reviewed and supported by the institutional review board at the Unfallkrankenhaus Berlin (*IRB-ukb-HNO-2015/01*) and has been conducted according to the principles expressed in the Declaration of Helsinki. Patient records and information were anonymized and de-identified prior to analysis.

### 2.1. Radiologic Evaluation

Determination of the insertion depth angle and cochlear size was performed using an Allura Xper FD20 system (Philips Medical Systems, Best, Netherlands) with a flat panel detector. The parameters of the system were as follows: entrance field of 22 cm, 274 mAs, 95 kV, 180° rotation, 241 projections, filter 0.90 mm Cu + 1.00 mm Al, and posteroanterior (PA). The focus panel distance was determined and constant over the entire rotation at a frequency of 30 pic/s. The 3D tomography was performed in the unsubtracted mode. From this volume data set, the temporal bones were secondarily enlarged (FoV of 100 mm), digitally stored and sent for 2D- and 3D-reconstruction to an external workstation (Extended Brilliance Workspace, Philips, Cleveland, USA). Two experienced surgeons and a neuroradiologist certified all radiological images postoperatively. For the scalar position of the electrode array, image acquisition and reconstruction were performed as described by Aschendorff et al. in 2007 [8].

### 2.2. Cochlear Size and the Insertion Depth Angle

**Figure 1:**
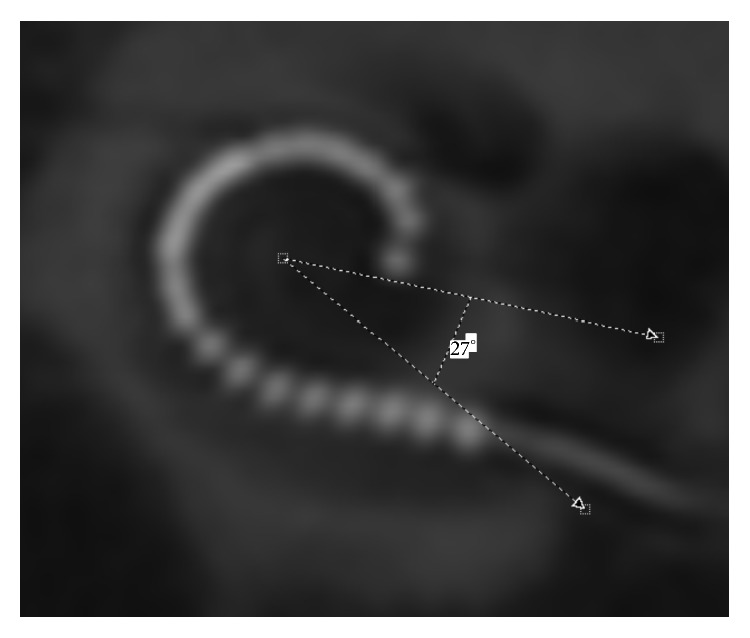
Exemplary measurement of the insertion depth angle. A reconstructed 2D “cochlear view” from the postoperative rotational tomography of patient 16. Insertion depth angle is 333°.

**Figure 2:**
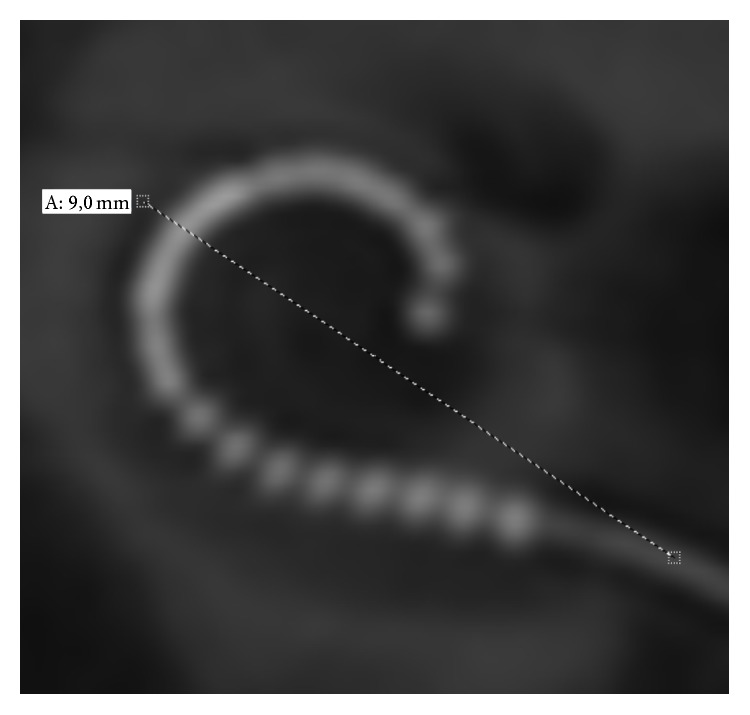
Exemplary measurement of the cochlear size. A reconstructed 2D “cochlear view” from the postoperative rotational tomography of patient 16. Cochlear size is 9 mm.

To quantify the cochlear size, the diameter of the basal turn was measured using the reconstructed cochlear view [11]. The diameter of the basal turn is measured from the center of the round window to the lateral wall, crossing the position of the helicotrema [19, 20]. This distance was chosen as it is sufficient for defining of the cochlear size [19]. For the determination of the insertion depth angle a reference line connecting the center of the round window and the center of the modiolus was defined at zero-degree angle. Another reference line from the center of the modiolus to the tip of the electrode array defines the angle that has to be subtracted or added if the electrode crosses the first reference line according to the consensus panel [12] (Figures 1 and 2).

### 2.3. Data Acquisition and NRT-Evaluation

NRT data were obtained intraoperatively under sterile conditions in all included patients. Software-based NRT recordings (Cochlear's Custom Sound 4.0) were used (Auto-NRT mode) to measure and evaluate the NRT thresholds (t-NRT). In each individual all electrodes were measured and recorded.

The NRT-ratios were calculated in each individual. This was determined by dividing the average NRT value from electrodes 18 and 16 in the apical part with the average NRT value from electrodes 8 and 6 in the basal part of the electrode array [17]. Statistical evaluation was performed using SPSS (Version 22.0; IBM Co., Armonk, NY, USA). In a second step modified NRT-ratios were correlated with the insertion depth angle and the cochlea size. NRT-ratios were calculated by shifting the selected electrode in the apical and basal part (Table 1).

## 3. Results

A certain CI electrode position within scala tympani was radiologically verified postoperatively in every patient. Pearson's product-moment correlation was performed to determine whether there was a relationship between the NRT-ratio and the insertion depth angle and cochlear size, respectively. For all variables, there were no outliers and the data was normally distributed, as assessed by a Shapiro-Wilk test (*p* > 0.05). The mean NRT-ratio was 0.90 (0.74–1.04, SD ± 0.08), the mean insertion depth angle was 359.11° (326°–400°, SD ± 24.09), and the mean basal cochlear diameter was 7.79 mm (7 mm–9 mm, SD ± 0.46). There was neither a correlation between the NRT-ratio and the insertion depth angle, *r*(24) = 0.056, *p* > 0.05, nor a correlation between the NRT-ratio and the cochlear size *r*(24) = −0.023, *p* > 0.05.

The NRT-ratio is a quotient between apical and basal CL levels. In the apical and basal part different electrodes were selected and shifted to calculate different NRT-ratios. These ratios were also correlated with the insertion depth angle and the cochlear diameter. A slight positive but not statistically significant correlation was seen between the NRT-ratios and the insertion depth angles (*r* < 0.3; *p* > 0.05) and a slight negative relationship was seen between the NRT-ratio and the cochlear size (*r* > −0.3; *p* > 0.05), respectively (Table 1).

Furthermore the relationship between the insertion depth angle and the cochlear size was investigated. Pearson's product-moment correlation showed a moderate negative correlation but not statistically significance with *r*(24) = −0.311, *p* > 0.05.

## 4. Discussion

Cochlear implantation is a safe and reliable procedure for the treatment of severe to profound sensorineural hearing loss and patients with residual hearing. The position of the electrode array in the cochlea is fundamental for the interaction between the implant and the cochlear neuronal structures. The NRT-ratio for the estimation of the intracochlear position of the CI is dependent on the intracochlear structures [17, 18]. In previous studies a variation of the NRT-ratio was seen for patients with an electrode position within scala tympani [17, 18]. The question arises if this variability is dependent on cochlear size or the insertion depth angle.

The vitality of neuronal structures and the amount of spiral ganglion degeneration among the cochlea are essential for the NRT patterns and the reliable NRT-ratio. Neurosensory, hereditary disorders such as Usher's syndrome [21], diseases with “nonhomogenous dead” ganglion cells such as superficial siderosis [22], or congenital rubella infection [23] could result in irregular t-NRT pattern. In patients with long-term deafness, the total number and viability of the spiral cell ganglions after this long period is not clear but can be assumed to be affected [24]. In such neural affected patients the NRT-ratio is less valid as in patients without degenerative neural impairments.

In our group of patients with a certain electrode position within the scala tympani the NRT-ratio shows variability between 0.74 and 1.04. As the electrically evoked cochlear action potential (ECAP) depends on the proximity of the electrode array and the spiral ganglion cells [16, 25, 26], the NRT thresholds depend as well on this distance. In the apex, the diameter of the cochlea is smaller and the electrode array converges to the spiral ganglion cells. Hence ECAP thresholds are lower in the apex than in the base [27]. Based on these findings an electrode with a greater insertion angle can be assumed to correlate with a smaller NRT-ratio. To our knowledge there are no published studies comparing the angular insertion depth with the electrophysiological pattern. In our study population, we do not see the tendency that greater insertion angles correlate with a smaller NRT-ratio. Nevertheless an unknown factor is still the lack of information about the vitality of spiral ganglion cells although NRT pattern was recorded regularly in all patients. We modified the NRT-ratio by regarding different electrode ranges in the apical and basal part (Table 1).

The cochlear diameter in our patient's population varied from 7.0 to 8.8 mm and is comparable to the literature [19, 28]. Similar to the insertion depth angle no relationship between the NRT-ratio and the cochlear size was found but a moderate not significant correlation between the insertion depth angle and the cochlear diameter was observed. This finding underlines the hypothesis by Escudé et al. [19] that, for perimodiolar arrays, the cochlear size may influence the insertion depth angle.

In comparison with our previous study [18] (mean NRT-ratio 0.88 (0.7–0.99) for patients with scala tympani position), where all implants were inserted via a cochleostomy anterior inferior to the round window, the mean NRT-ratio is a little higher in the present study as implants were inserted via the round window or an enlarged RW. CI electrode arrays inserted via a cochleostomy have deeper insertion depth angles than those inserted via the round window, even if inserted to the same marker's position [29].

## 5. Conclusion

The NRT-ratio allows the distinction between a regular and a scalar changing position of perimodiolar CI electrodes for the surgeon. The insertion depth angle and the cochlear size have no statistically significant influence on the NRT-ratio.

## Figures and Tables

**Table 1:** Overview of the correlation between the NRT-ratio, the insertion depth angle, and the cochlear size.

Patient	IDA in °	CS in mm	NRT-ratio original (16–18/5–7)	NRT-ratio mod. 1 (16–18/3–5)	NRT-ratio mod. 2 (16–18/4–6)	NRT-ratio mod. 3 (16–18/2–4)	NRT-ratio mod. 4 (18–20/2–4)	NRT-ratio mod. 5 (18–20/3–5)	NRT-ratio mod. 6 (18–20/4–6)	NRT-ratio mod. 7 (18–20/5–7)
1	328	7.6	0.96	0.87	0.91	0.87	0.91	0.93	0.97	1.05
2	333	8.2	0.84	0.81	0.84	0.77	0.76	0.79	0.82	0.83
3	343	7.8	1.04	0.95	0.97	0.87	0.84	0.88	0.9	0.88
4	373	8	0.91	1.08	1.11	1.02	1	1.07	1.1	1.1
5	326	8	0.93	0.91	0.92	0.89	0.88	0.89	0.9	0.91
6	332	8.3	0.75	0.7	0.73	0.71	0.74	0.78	0.83	0.87
7	392	7.9	1	1.06	1.03	1.09	1.08	1.09	1.06	0.99
8	367	7.3	0.89	0.84	0.89	0.8	0.81	0.87	0.93	0.95
9	400	8	0.95	0.94	0.96	0.88	0.86	0.91	0.93	0.93
10	353	7.2	0.91	0.87	0.91	0.8	0.76	0.81	0.85	0.86
11	392	7.8	0.84	0.85	0.85	0.82	0.79	0.81	0.81	0.79
12	373	7.2	0.77	1	0.98	0.92	0.86	0.89	0.87	0.84
13	368	7.6	0.88	0.83	0.87	0.79	0.77	0.79	0.84	0.85
14	332	7.6	0.98	0.99	1.01	1	1	1.01	1.03	1
15	370	8.8	0.87	0.87	0.89	0.82	0.81	0.82	0.84	0.83
16	333	9.0	0.91	0.84	0.88	0.81	0.83	0.88	0.92	0.95
17	381	7.4	0.86	0.88	0.88	0.82	0.81	0.84	0.84	0.82
18	356	8.4	0.8	0.73	0.77	0.64	0.58	0.6	0.63	0.66
19	356	8.4	1.04	1	1.04	0.97	0.95	0.97	1	1.01
20	358	8.2	0.91	0.98	0.97	0.91	0.93	0.98	0.97	0.91
21	332	7.8	0.74	0.7	0.74	0.68	0.73	0.78	0.82	0.83
22	320	8.7	0.95	0.89	0.93	0.8	0.77	0.82	0.86	0.88
23	380	7.4	0.98	0.96	0.99	0.88	0.88	0.97	1	0.99
24	388	7.5	0.92	0.92	0.96	0.82	0.79	0.87	0.9	0.87
25	382	7	0.79	0.7	0.75	0.67	0.71	0.75	0.8	0.85
26	351	7.4	0.9	1	0.96	1.01	1.05	1.08	1.03	0.97

*r* (IDA versus NRT)	—	—	0.056	0.286	0.218	0.201	0.113	0.196	0.111	−0.024
*p* value^*∗*^	—	—	>0.05	> 0.05	>0.05	>0.05	>0.05	>0.05	>0.05	>0.05

*r* (CS versus NRT)	—	—	−0.023	−0.311	−0.103	−0.208	−0.217	−0.224	−0.273	−0.214
*p* value^*∗*^	—	—	>0.05	>0.05	>0.05	>0.05	>0.05	>0.05	>0.05	>0.05

IDA: insertion depth angle; CS: cochlear size; NRT: neural response telemetry.

^*∗*^ *p* values were calculated using Pearson's product-moment correlation.
